# Bidirectional Dynamics Between Stress-Reactive Rumination and Negative Affect: Evidence From a Multimethods Study in Individuals With High Trait Anxiety

**DOI:** 10.1155/da/2503361

**Published:** 2025-09-02

**Authors:** Zhu Qingzi, Peng Lanxin, Niu Lijing, Zeng Yuanyuan, Chen Xiayan, Chen Zini, Dai Haowei, Zhang Ruibin

**Affiliations:** ^1^Laboratory of Cognitive Control and Brain Healthy, Department of Psychology, School of Public Health, Southern Medical University, Guangzhou, China; ^2^Department of Psychiatry, Zhujiang Hospital, Southern Medical University, Guangzhou, China; ^3^Guangdong-Hong Kong-Macao Greater Bay Area Center for Brain Science and Brain-Inspired Intelligence, Guangdong-Hong Kong Joint Laboratory for Psychiatric Disorders, Guangdong Basic Research Center of Excellence for Integrated Traditional and Western Medicine for Qingzhi Diseases, Guangzhou, Guangdong, China

**Keywords:** ecological momentary assessment, negative affect, stress-reactive rumination, trait anxiety

## Abstract

**Background:** Stress-reactive rumination (SR)—the tendency to repetitively think about stressors—has been proposed as a key cognitive mechanism linking trait anxiety to persistent negative affect (NA). However, the dynamic and context-dependent nature of this relationship remains unclear. This study employed a multimethod experimental design to investigate the manifestation of SR in individuals with high trait anxiety (HTA) across different contexts and to examine whether SR and NA demonstrate a bidirectional predictive relationship over time.

**Methods:** A total of 62 participants (31 with high and 31 with low trait anxiety (LTA), respectively) completed a 14-day ecological momentary assessment (EMA) four times/day to record dynamic fluctuations in SR and NA over time in daily life. Afterward, participants underwent the Trier social stress test (TSST) to examine SR and NA responses under acute laboratory stress. Cross-lagged models assessed temporal associations between SR and NA in both contexts.

**Results:** EMA data showed that individuals with HTA reported significantly higher SR and NA than their low anxiety counterparts (*p* < 0.001). Cross-lagged analyses revealed a significant bidirectional predictive relationship between SR and NA, although this relationship was present only in the HTA group (SR → NA: *b* = 0.159, *p* < 0.001; NA → SR: *b* = 0.072, *p* = 0.038). In the laboratory, SR and NA were successfully induced by stress, particularly in the HTA group (*p* < 0.001), but cross-lagged effects were not observed.

**Conclusions:** In individuals with HTA, SR demonstrates both persistence and context-dependent reactivity. The observed reciprocal dynamics between SR and NA in real-life settings underscore the role of attentional control deficits in maintaining emotional dysregulation. Interventions targeting SR may disrupt this maladaptive cycle and reduce anxiety vulnerability.

## 1. Introduction

Anxiety-related disorders are among the most prevalent and disabling mental health conditions worldwide [1, 2], and a core feature across diagnostic boundaries is the experience of persistent and dysregulated negative affect (NA). Trait anxiety, a stable predisposition to respond with heightened fear and worry, has been consistently associated with elevated NA, particularly in the context of daily stress [3]. Moreover, individuals with high trait anxiety (HTA) are at increased risk of developing anxiety and other psychiatric disorders over time [4]. Yet, while trait anxiety increases vulnerability to negative emotional experiences, the psychological mechanisms that drive this emotional persistence remain incompletely understood.

According to the control–process model [5], rumination consumes existing cognitive resources and impairs problem-solving abilities, thereby further exacerbating and sustaining anxiety in individuals with HTA who already exhibit attentional control deficits [6]. Existing research has found that stress-reactive rumination (SR) can independently predict anxiety symptoms [7]. Unlike general rumination, which involves passive, repetitive thinking about one's own distressing symptoms and their causes, meanings, and consequences [8], SR is triggered by specific adverse events or stressful situations and emerges rapidly, characterized by temporal immediacy and situational specificity [9]. These qualities make it a more proximal driver of anxious arousal. When a stressor occurs, SR quickly monopolizes the attention, problem-solving, and emotion-regulation resources needed to cope with anxiety, thereby, amplifying anxious reactions in the short term. Individuals with HTA typically display an attentional bias toward negative stimuli and impaired attentional disengagement from negative emotional stimuli [6, 10]. These interrelated cognitive deficits (heightened sensitivity to NA, diminished attentional control, and impaired emotion regulation) [6, 11, 12] render them more susceptible to falling into SR after stressful events. Furthermore, due to having insufficient cognitive resources to disengage from or regulate NA, individuals with HTA are more prone to entering a vicious cycle of rumination and NA.

Although the evidence above suggests a possible bidirectional relationship between SR and NA, the manifestation of SR in the HTA group and the dynamic nature and context-dependency of this relationship remain unclear. Specifically, is SR a stable response tendency or a stress-induced state in HTA individuals? Does its interaction with NA differ between real-world and laboratory settings? Previous research—primarily relying on cross-sectional or single-method designs—that answers questions such as these has been limited.

To clarify this issue, the present study employed a multimethod experimental design combining an ecological momentary assessment (EMA) approach [13] and laboratory procedures. EMA is an effective tool for assessing repetitive negative thinking [14]. It collects real-time behavioral and affective data from individuals in their natural environments, reflecting more authentic emotional states [15], while also enhancing ecological validity [16] and reducing recall bias [17]. Using EMA in the present study allowed us to monitor SR in the HTA group during daily stressful situations. In the laboratory, stress-induction methods can be used to provoke SR thoughts [18, 19]. This study used the Trier social stress test (TSST) [20] to activate SR [21, 22], thereby, investigating SR elicitation in the HTA group under laboratory-induced stress. Combining EMA with laboratory stress induction addresses the limitations of single-timepoint laboratory measurements in capturing SR and the dynamic fluctuations of the SR–NA relationship [23]. This approach examines SR manifestation in the HTA group from two dimensions: on the one hand, if SR reflects a stable cognitive vulnerability (e.g., deficits in attentional control), the HTA group should consistently show higher SR levels in both daily life (EMA) and the laboratory; on the other hand, if SR is a context-dependent response, the HTA group should exhibit a significantly greater increase in SR following TSST-induced stress compared to the low trait anxiety (LTA) group. Moreover, the bidirectional dynamic relationship was tested using cross-lagged models.

This study innovatively combined EMA with a classic laboratory stress-induction paradigm, the TSST, to address two core research questions: First, does SR persist or is it only induced by stress in individuals with HTA? Second, is there a significant bidirectional relationship between SR and NA in HTA individuals? We hypothesized that SR in HTA individuals can be induced by stress, persists over time, and exhibits a clear bidirectional interaction with negative emotional experiences.

## 2. Methods

### 2.1. Participants

A total of 500 students from Southern Medical University underwent initial screening. Participants were excluded based on three criteria: (1) the presence of depressive symptoms (Beck Depression Inventory II [BDI-II] score ≥14 points or Patient Depression Questionnaire–9 [PHQ-9] score ≥10 points) [24, 25]; (2) diagnosis of chronic/acute physical illnesses; (3) prior participation in TSST experiments. Anxiety traits were evaluated using the state–trait anxiety inventory-trait subscale (STAI-T) [26], with participants stratified into high anxiety (top 27%, STAI-T score ≥50 points) and low anxiety (bottom 27%, STAI-T score ≤36 points) groups following established methodology [27–29]. The final sample included 62 participants (high anxiety: *n* = 31, 7 males/24 females; low anxiety: *n* = 31, 11 males/20 females), with a mean age of 21.980 years (SD = 1.509). Seven participants (four males and three females) withdrew due to scheduling conflicts during TSST implementation. The study protocol received ethical approval from Southern Medical University's ethics committee. All participants provided written informed consent after receiving a full explanation of experimental procedures and received monetary compensation upon study completion.

### 2.2. Procedure

**Figure 1:**
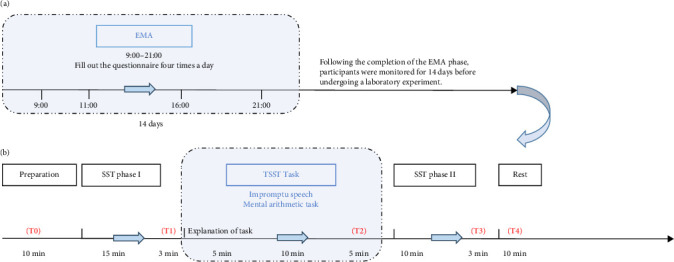
Research flowchart. (a) Ecological momentary assessment (EMA); (b) laboratory protocol for inducing stress-reactive rumination. The laboratory assessments were administered at three time points: before the experiment (T0), after the TSST (T2), and after SST phase II (T3). Saliva cortisol samples were collected at five time points: before the experiment (T0), after SST phase I (T1), after the TSST (T2), after SST phase II (T3), and 15 min after the rest period (T4). NA, negative affect; SR, stress-reactive rumination; SST, stop signal task; TSST, Trier social stress test.

This study employed a multimethod approach integrating EMA with laboratory-based stress induction to investigate the dynamics between SR and NA. The procedure included two phases: First, EMA captured participants' stress-related cognitive and emotional responses in daily life; then, TSST induced acute stress under controlled conditions to assess corresponding changes in SR and NA. The following sections describe the EMA and laboratory procedures (Figure 1).

#### 2.2.1. EMA

Prior to initiating the EMA, participants received standardized protocol training. The EMA protocol spanned 14 days, requiring participants to complete four daily assessments (at 09:00, 11:00, 16:00, and 21:00 h) via a mobile research platform. Each assessment lasted approximately 3 min and required submission within 30 min of notification.

The EMA instrument included four modules, as follows: (1) perceived stress (PS): A 100-mm visual analog scale (VAS; anchors: “no stress” [0] to “extreme stress” [100]) was used to assess cumulative stress since the last assessment; (2) perceived stress impact (PSI): a VAS (0–100) was used to evaluate the perceived impact of stressors, along with free-text entries about the stressors (not included in the analysis); (3) SR: 10 VAS items were used to assess five dimensions (replay, persistence, contemplation, criticism, and negativity) [18, 30, 31]; (4) NA: The NA subscale of the Positive and NA schedule [32], containing 10 items (irritable, alert, ashamed, inspired, nervous, determined, attentive, jittery, active, and afraid), was adopted, and participants rated each item on a five-point likert scale ranging from 1 (“not at all”) to 5 (“extremely”) points.

#### 2.2.2. Laboratory Paradigm for Activating SR (TSST)

Following EMA completion, participants underwent 14-day monitoring before laboratory assessment. To standardize cortisol measurements [33], all participants adhered to the following three pretest requirements: (1) 2-h abstinence from exercise and nonwater drinks; (2) fasting; (3) rescheduling of any sessions coinciding with menstrual phases. Testing occurred during 12:30–18:00 h to control diurnal cortisol variation. Upon arrival, participants received instructions about a “cognitive evaluation” before consent. Baseline procedures included, (1) demographic collection; (2) salivary cortisol sampling; (3) multidimensional assessments mirroring EMA protocols (laboratory multidimensional stress [LMS], SR, and NA), with adaptations, as follows: SR focused on interview-speech stressors, while LMS used five validated dimensions (PS, unpleasantness, difficulty, irritation, fear; Table S1) to comprehensively assess subjective stress in the laboratory setting.

The experimental procedure involved three phases. First, participants completed a 15-min stop signal task (SST Phase I; SST data were collected but not analyzed). Then, the TSST was administered, consisting of three components: 5 min of speech preparation, 5 min of impromptu presentation, and a 5-min mental arithmetic task requiring serial subtraction of 13 from 1022, all performed before a gender-balanced evaluation panel. Immediately following the TSST, participants rated their LMS, SR, and NA using a VAS. Third, a post-stress SST (Phase II; 15 min) was conducted, followed by a standardized 10-min recovery period. The session concluded with a comprehensive debriefing protocol to mitigate residual stress effects.

### 2.3. Data Analysis

#### 2.3.1. EMA Data Analysis

First, independent-samples *t* tests were conducted on EMA data to examine how SR manifested in the HTA group under everyday conditions, while simultaneously comparing group differences in PS, PSI, and NA.

Second, to estimate the effect of group membership (categorical variable: 1 = HTA group; 2 = LTA group) on PS, as well as the relationship between PS, SR, and NA, random-intercept multilevel modeling was conducted using R (v 4.1.2; R Foundation for Statistical Computing, Vienna, Austria; https://www.R-project.org/) along with packages such as lme4, DataCombin, and sjPlot. In the multilevel structure, assessments at each time point (Level 1) were nested within participants (Level 2). All continuous predictor variables were person-mean–centered to distinguish within-person from between-person effects.

In the first step of the multilevel analysis, the total scores for PS and SR from the EMA data were used as dependent variables to explore the effect of group membership with following equation:

(1) $$\begin{array}{c} Y_{ij}=\beta_{00}+\beta_{01}\left(\text{Group}\right)+\beta_{02}\left({\text{age}}_{j}\right)+\beta_{03}\left({\text{gender}}_{j}\right)+\beta_{04}\left({\text{times}}_{ij}\right)+u_{0j}+r_{ij}, \end{array}$$

where *Y _ij_* represents the total score of individual *j* at time point *i* for each evaluation component—specifically, the scores for SR and NA. The within-subject effect is modeled at Level 1, where *j* denotes each participant and ii represents each assessment time point. The *β* coefficients indicate the intercept, the main effect of group, the effect of gender, and the effect of the Level 1 covariate (time). Random effects are represented by the intercept *u*_0*j*_, and *r*_*ij*_ denotes the residuals for individual *j* at time point *i*.

Given that SR theoretically arises in response to stress, the second step aimed to investigate the impact of PS on SR while accounting for group differences. This model included both trait and PS levels as predictors, along with their interaction effects, with following equation:

(2) $$\begin{array}{c} Y{\left(\text{SR}\right)}_{ij}=\beta_{00}+\beta_{01}\left(\text{Group}\right)+\beta_{02}\left({\text{PS}}_{j}\right)+\beta_{03}\left({\text{Group}}^{*}{\text{PS}}_{j}\right)+\beta_{04}\left({\text{gender}}_{j}\right)+\beta_{05}\left({\text{age}}_{j}\right)+\beta_{10}\left({\text{times}}_{ij}\right)+u_{0j}+r_{ij}, \end{array}$$

where *Y* (*SR*)_*ij*_ represents the total score of SR for the *j* individual at the *i* time point. Within-subject effects are modeled at the first level, with each subject (subscript *j*) representing the value for each assessment (subscript *i*). The *β* coefficients represent the intercept, the main effects of the predicted group, gender effects, and the effect of the first-level covariate (time). Random effects are represented by the intercept *u*_0*j*_, and *r*_*ij*_ represents the residual for the *j* individual at the *i* time point.

To examine emotional dynamics between the two groups, lagged variables (*t−*1 and *t−*2 emotional levels) were calculated, with the first observation of each day set as missing. A random-intercept multilevel model was constructed, with age and gender included as covariates. The complete models are presented with the following equations:

(3) $$\begin{array}{c} Y{\left(\text{SR/PS}\right)}_{ij}=\beta_{00}+\beta_{01}\left(\text{Group}\right)+\beta_{02}\left({\text{SR}}_{t-1}/{\text{PS}}_{\left(t-1\right)j}\right)+\beta_{03}\left({\text{SR}}_{t-1}/{{\text{PS}}_{\left(t-1\right)j}}^{*}\text{Group}\right)+\beta_{04}\left({\text{gender}}_{j}\right)+\beta_{05}\left({\text{age}}_{j}\right)+u_{0j}+r_{ij}, \end{array}$$

(4) $$\begin{array}{c} Y{\text{(SR/PS)}}_{ij}=\beta_{00}+\beta_{01}\left(\text{Group}\right)+\beta_{02}\left({\text{SR}}_{t-2}/{\text{PS}}_{\left(t-2\right)j}\right)+\beta_{03}\left({\text{SR}}_{t-2}/{{\text{PS}}_{\left(t-2\right)j}}^{*}\text{Group}\right)+\beta_{04}\left({\text{gender}}_{j}\right)+\beta_{05}\left({\text{age}}_{j}\right)+u_{0j}+r_{ij}, \end{array}$$

where *Y*_*ij*_ represents the total score of SR and PS for the *j* individual at the *i* time point. Within-subject effects are modeled at the first level, with each subject (subscript *j*) representing the value for each assessment (subscript *i*). The *β* coefficients represent the intercept, the main effects of the predicted group, gender effects, and the effect of the first-level covariate (time). Random effects are represented by the intercept *u*_0*j*_, and *r*_*ij*_ represents the residual for the *j* individual at the *i* time point.

**Figure 2:**
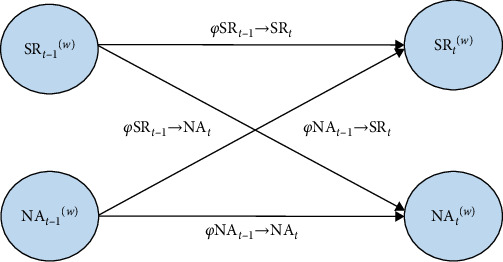
A multilevel cross-lagged model of temporal association between stress-reactive rumination (SR) and negative affect (NA).

Finally, to thoroughly examine the dynamic interplay between SR and NA in everyday life contexts, cross-lagged analyses were conducted using Mplus version 8.5 (Muthen & Muthen, Los Angeles, CA, USA). Dynamic structural equation modeling was employed, with models run using noninformative Bayesian estimation, following prior studies [34–37]. The cross-lagged relationship between SR and NA is illustrated in Figure 2.

Because SR and NA at the previous time point (*t ****−****1*) predicted their respective values and each other at the current time point (*t*), a random-intercept model was used to analyze both autoregressive (self-path) and cross-lagged paths. Similarly, the first time point of each day was set as missing. Within-subject components were represented at Level 1, describing the SR and NA of individual *j* at time *t*, as shown in following equations:

(5) $$\begin{array}{c} Y{\left(\text{SR}\right)}_{tj}=\mu {\text{SR}}_{j}+\phi_{1j}\left({\text{SR}}_{jt-1}^{\left(w\right)}\right)+\phi_{3j}\left({\text{NA}}_{jt-1}^{\left(w\right)}\right)+\zeta_{1jt}, \end{array}$$

(6) $$\begin{array}{c} Y{\left(\mathrm{NA}\right)}_{tj}=\mu {\text{NA}}_{j}+\phi_{2j}\left({\text{NA}}_{jt-1}^{\left(w\right)}\right)+\phi_{4j}\left({\text{SR}}_{jt-1}^{\left(w\right)}\right)+\zeta_{2jt}, \end{array}$$

where *Y*_*tj*_ represents the total score of each assessment for the *j* individual at the *t* time point, including the total scores for SR and NA. _*μ*_SR_*j*_ and _*μ*_NA_*j*_ represent the fixed effects of SR and NA for individual *j*. The autoregressive coefficients *φ*_1 *j*_ and *φ*_2 *j*_ represent the influence of the variables at time *t−*1 on themselves at time *t*. The cross-regression coefficients *φ*_3 *j*_ and *φ*_4 *j*_ represent the mutual influence of the variables at time *t−*1 on each other at time *t*, with (w) indicating internal estimation. The random error terms are represented by *ζ*_1jt_ and *ζ*_2*jt*_.

#### 2.3.2. Laboratory Data Analysis

Saliva samples were collected during the experiment using Salivette tubes (REF51.1534.500; Sarstedt AG & Co., Nümbrecht, Germany). The collected saliva samples were stored in a −78°C freezer. Prior to analysis, the samples were thawed and centrifuged at 3000 rpm for 10 min. Salivary cortisol concentrations were measured using an electrochemiluminescence immunoassay. For cortisol data, a 2 × 5 repeated-measures analysis of variance (ANOVA) was conducted to assess the validity of the stress-induction procedure, with five time points as the within-subjects variables and group (two levels: HTA and LTA) as the between-subjects variable. To explore inter-group differences in laboratory stress response, an independent-samples *t* test was subsequently performed under each time point.

Laboratory data were analyzed using SPSS version 26 (IBM Corp., Armonk, NY, USA) to examine SR in the HTA group under laboratory conditions. First, a 2 × 3 repeated-measures ANOVA was conducted, with group (HTA vs., LTA) as the between-subjects variable and time point (T0, T2, and T3) as the within-subjects variable. Subsequently, paired-samples *t* tests were performed on the questionnaire data collected at T0, T2, and T3. To analyze inter-group differences at each time point, independent-samples *t* tests were conducted on LMS scores, SR scores, and NA scores across the three time points. Since the first item in the LMS—PS—is the core indicator of laboratory stress assessment and aligns with the daily EMA assessment content, this item was also analyzed as a separate variable. Note, to distinguish between them, the total score of the LMS will be referred to as “LMS total score” in the following text, while the score for the first item will be referred to as “laboratory PS.”

Finally, cross-lagged model analyses analogous to those applied to the EMA data were conducted to investigate the dynamic relationship between SR and NA under laboratory conditions, with the distinction that variables were measured at only three time points (T0, T2, and T3) and denoted as SR (SR_1_, SR_2_, and SR_3_) and NA (NA_1_, NA_2_, and NA_3_). To remove the influence of the baseline (T0), difference scores were calculated and used in the analysis: △SR_2_ (△SR_2_ = SR_2_ − SR_1_), △SR_3_ (△SR_3_ = SR_3_ − SR_1_), △NA_2_ (△NA_2_ = NA_2_ − NA_1_), and △NA_3_ (△NA_3_ = NA_3_ − NA_1_).

#### 2.3.3. Correlation Analysis of EMA and Laboratory Questionnaire Data

To investigate the similarities and differences between the laboratory task and the EMA data—and to assess the external validity of the laboratory task—Pearson correlations were computed between the mean and standard deviation values of the first item in the LMS (i.e., PS), as well as SR and NA, with the corresponding mean and standard deviation values from the EMA measurements [38].

## 3. Results

### 3.1. Sample Demographic Characteristics

The HTA group and LTA group showed no significant differences in age or gender distribution. However, the HTA group scored significantly higher than the LTA group on the trait anxiety scale, PHQ-9, and BDI-II (Table S2).

### 3.2. Dynamic Links Between SR and NA Captured by EMA

A total of 62 participants completed 3313 assessments (out of a theoretical total of 3472). The average number of responses per individual was 53.445 (SD = 2.558; range, 45–56). In the HTA group, the average number of responses was 53.484 (SD = 2.308), while, in the LTA group, the average number of responses was 53.387 (SD = 2.825). There was no statistically significant difference between the two groups in the number of responses (*t* = 0.148, *p* = 0.883). An independent-samples *t* test on the questionnaire scores revealed significant differences between the two groups in the total scores for PS, PSI, SR, and NA (*p* < 0.001), with the HTA group scoring higher on all sections than the LTA group (Table S3).

#### 3.2.1. Temporal Characteristics of SR

The random-intercept multilevel model analysis revealed that the HTA group exhibited significantly stronger SR after controlling for demographic variables (*p* < 0.05). PS level (*b* = 8.110, SE = 0.300, *p* < 0.001) and its interaction with group membership (*b* = 2.060, SE = 0.210, *p* < 0.001) emerged as core predictors of SR, replacing the direct effect of group membership (Table S4). Lag analyses revealed that both PS and SR at *t*−1 and *t−* significantly predicted current levels (*p* < 0.001; Table S5). The interaction between group and SR was only significant at the *t* time point (*b* = −0.110, SE = 0.040, *p* = 0.007). In the *t* model, group significantly predicted current SR (*b* = −57.810, SE = 27.540, *p* = 0.036). However, the autoregressive effect of PS levels showed no between-group differences (*p* > 0.05; Table 1).

#### 3.2.2. The Reciprocal Prediction Between SR and NA in the HTA Group

**Figure 3:**
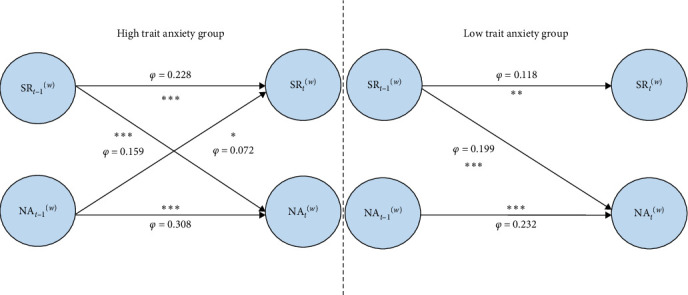
The autoregressive and cross-lagged parameters of SR and NA in different trait anxiety groups. Only paths with *p*-values < 0.05 are shown. The thickness of each line visually represents the strength of statistical significance—thicker lines correspond to lower *p*-values, indicating stronger effects. *⁣*^*∗∗∗*^*p* < 0.001, *⁣*^*∗∗*^*p* < 0.01, *⁣*^*∗*^*p* < 0.05.

Results from the cross-lagged model demonstrated that both groups exhibited significant autoregressive effects for SR and NA (Table 2) and that SR at the previous time point significantly predicted NA at the subsequent time point (Table 2). In the HTA group, NA at the previous time point also significantly predicted SR at the subsequent time point (*b* = 0.072,SD = 0.040, *p* = 0.038), whereas, in the LTA group, this effect was not significant (*b* = 0.027, SD = 0.037, *p* = 0.240) (Figure 3).

### 3.3. Laboratory Evidence for Acute Stress-Induced Rumination

#### 3.3.1. Successful Stress Induction and Differential Emotional Responses

**Figure 4:**
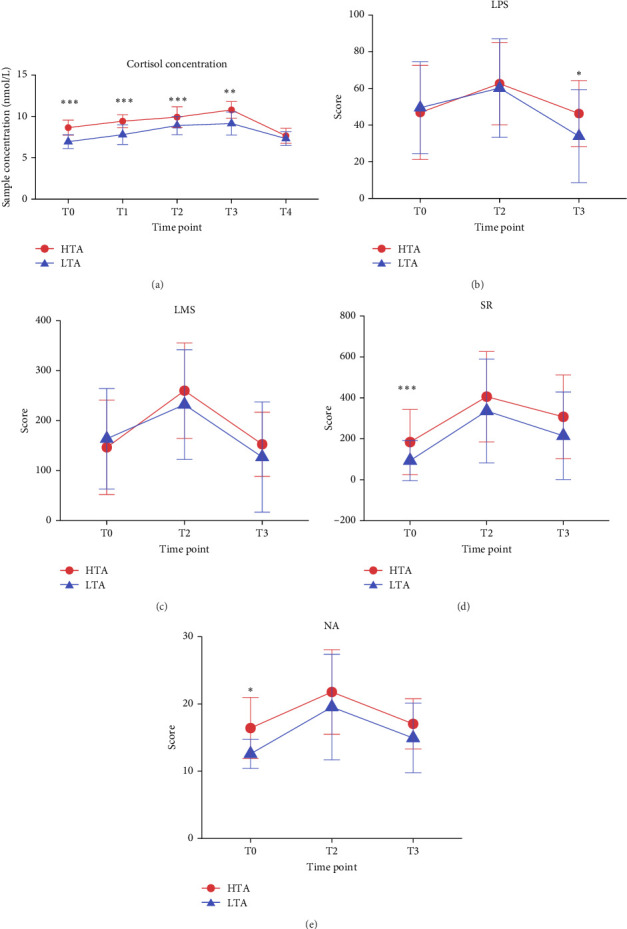
Acute stress-induced cortisol and emotional responses. Line plots showing the changes in cortisol concentration (a), LPS = laboratory perceived stress (b), LMS = laboratory multidimensional stress (c), SR = stress-reactive rumination (d), and NA = negative emotions (e) across different time points in the laboratory. The red line represents the high trait anxiety (HTA) group, while the blue line represents the low trait anxiety (LTA) group. Error bars indicate standard deviation. *⁣*^*∗∗∗*^*p* < 0.001, *⁣*^*∗∗*^*p* < 0.01, *⁣*^*∗*^*p* < 0.05 indicate significant differences between groups.

The *t* test and ANOVA results on the cortisol data indicate that the stress induction was successful (Table S5 and Figure 4). Paired-samples *t* test results for the questionnaire data revealed significant differences in LMS, laboratory PS, SR, and NA between T2 and baseline (T0), as well as between T2 and T3 (*p* < 0.001), indicating that the TSST successfully induced SR. Independent-samples *t* test results showed no significant difference in LMS at baseline (T0) between the HTA and LTA groups; however, significant group differences were observed in SR (*p* = 0.014) and NA (*p* < 0.001). At T3, laboratory PS levels also differed significantly between the two groups (*p* = 0.042) (Figure 4). Interaction effect analysis indicated a significant main effect of time, with laboratory PS being the only measure showing a significant interaction effect between groups (*F* = 3.348, *p* = 0.043, *η* = 0.114). Further simple effects analysis revealed that, in the HTA group, T2 was significantly higher than both T0 (*p* = 0.034) and T3 (*p* < 0.001), while, in the LTA group, T3 was significantly lower than both T1 (*p* = 0.008) and T2 (*p* < 0.001) (Table S5).

#### 3.3.2. Patterns of SR Under Stress-Induced Conditions

In the cross-lagged model analysis of the HTA group (*n* = 27) and the LTA group (*n* = 28), both groups exhibited significant autoregressive effects, but no cross-lagged effects were found (Table 2). Specifically, in the LTA group, a significant autoregressive effect was observed for NA (△NA_2_ → △NA_3_: *b* = −0.624, *SD* = 0.193, *p* < 0.001). In contrast, the HTA group displayed significant autoregressive effects not only for NA (△NA_2_ → △NA_3_: *b* = 0.592, *SD* = 0.098, *p* < 0.001) but also, notably, for SR (△SR_2_ → △SR_3_: *b* = 0.506, *SD* = 0.134, *p* < 0.001).

### 3.4. Correlations Between EMA and Laboratory Questionnaire Data

In the correlational analysis between EMA data and the questionnaire data from this experiment, the laboratory's average PS (*r* = 0.409, *p* = 0.002), average SR (*r* = 0.288, *p* = 0.033), average NA (*r* = 0.412, *p* = 0.002), and the standard deviation of NA (*r* = 0.268, *p* = 0.048) were significantly correlated with the corresponding EMA indicators (Figure S1 in the), whereas the other standard deviations showed no significant correlations. This may suggest that the levels of PS and SR induced in the laboratory cannot be directly compared to the levels of PS induced by real-life stressors. However, when correlation analyses were conducted separately for each group, the correlation remained significant in the LTA group but was no longer significant in the HTA group.

## 4. Discussion

This study employed EMA in combination with the laboratory TSST to validate the hypothesis that SR exhibits both state-like and trait-like characteristics, with trait-like features being more pronounced in HTA individuals. Crucially, our analyses revealed a significant bidirectional predictive relationship between SR and NA exclusively in the HTA group.

### 4.1. SR and Its Reciprocal Link With NA

The present study found distinct manifestations of SR in individuals with HTA across daily-life and laboratory settings. Specifically, during the 14-day EMA assessment, the HTA group exhibited significantly stronger SR than the LTA group. This pattern was similarly observed during the laboratory baseline session. However, following successful stress induction via the TSST experimental task, although SR levels were higher in the HTA group, no significant difference in SR levels emerged between the two groups. Furthermore, results from the laboratory cross-lagged model showed that current SR levels were strongly dependent on the preceding state in both groups (from T2 to T3). Considering the laboratory baseline session as a random point in daily life, these results may indicate that SR in individuals with HTA can be triggered by stress and exhibits context-dependency, while, over a longer temporal span, it manifests as a persistent cognitive pattern unaffected by contextual variation (i.e., SR levels remained significantly higher in both EMA and laboratory baseline assessments). This finding aligns with the high frequency and persistent nature of ruminative thinking observed in studies of individuals with depression [39]. It is important to note that the laboratory component of this study involved limited sampling points, which may have hindered the capture of short-term fluctuations in SR. Future research needs to incorporate more sampling points to adequately evaluate SR manifestations within laboratory contexts.

In the analysis of the relationship between SR and NA in both groups, a bidirectional predictive relationship was found only in the HTA group. In contrast, individuals with LTA demonstrated some resistance to the influence of NA on SR. The separation observed in the cross-lagged model results for the two groups can be explained through the control–process model. When individuals with HTA experience stress or negative events, they engage in repetitive thinking patterns, that is, SR. Initially, rumination may have an adaptive function, focusing attention on the problem at hand and attempting to find a solution, which helps reduce NA. However, if this reflective process does not translate into effective problem-solving strategies, it becomes a repetitive, rigid thought pattern. This rigid rumination not only continues to reinforce NA but also weakens problem-solving abilities, making it difficult for individuals to disengage from NA and creating a self-sustaining vicious cycle. Furthermore, this vicious cycle may also be related to attentional control. The attentional control theory [40] posits that anxiety impairs the ability to control the suppression of irrelevant or distracting information. In other words, whereas nonanxious individuals can flexibly shift their attention with changing stimuli, individuals with HTA experience reduced control over distraction suppression and response inhibition to mental set biases under negative emotional induction. This lack of control may contribute to the formation and maintenance of high anxiety [41, 42]. The relationship between attention, NA, and rumination can be explained by the attentional scope model, which suggests that NA narrows the attentional scope, reducing the range of thoughts, perceptions, and actions activated in working or long-term memory, thus, increasing the likelihood of rumination. In contrast, positive emotions expand the attentional scope and decrease the likelihood of rumination [43]. Therefore, the attentional bias toward emotional valence in HTA individuals may serve as a key moderating variable in the bidirectional relationship between SR and NA. This also accounts for the trait-like tendency of SR observed within this population. Future research could focus on targeted interventions for SR in HTA individuals to break this vicious cycle.

### 4.2. Contextual Modulation of Rumination and Affective Responses by Trait Anxiety

The present study revealed distinct manifestations of SR in individuals with HTA across daily life and laboratory settings. Specifically, its findings highlight the central role of contextual factors in shaping the relationship between trait anxiety, SR, and NA. A noteworthy general observation was that both the HTA and LTA groups reported no significant differences in PS intensity, whether assessed in daily life or the laboratory. However, laboratory-measured salivary cortisol levels were significantly higher in the HTA group at multiple time points (T0–T3). This aligns with prior research indicating that individuals with HTA and LTA often do not differ significantly in subjective stress reports; instead, differences typically emerge only when these assessments are combined with other physiological or neurobiological measures [44–46]. This dissociation between subjective stress perception and objective physiological reactivity may reflect a maladaptive mechanism in HTA individuals. Frequent engagement in SR might lead to habituation to subjective stress experiences, albeit at the cost of heightened physiological reactivity and emotional inertia. Support for this comes from the random intercept multilevel model results: even when reporting similar levels of PS, HTA individuals exhibited a greater propensity for SR in response to the same stressful events.

Given that SR is a repetitive thinking pattern characterized by negative mentation following stressful events [47, 48], it is pertinent to discuss the expression and relationship among stress, SR, and NA in a comparison between the HTA and LTA groups. In the laboratory, after inducing SR using the same standardized strong stressor (TSST), the difference in NA levels between the two groups also disappeared (consistent with the SR results, there was a significant difference at the laboratory baseline, which disappeared following stress induction). However, EMA data from daily life revealed a crucial difference: HTA individuals consistently showed higher levels of SR in naturalistic settings when confronted with diverse stressors that, while potentially lower in intensity, were more personally relevant. This may suggest that differences in individual sensitivity to specific types of stressors could be a significant factor underlying SR level disparities. When considering PS as a common predictor of SR, such significantly predicted SR levels, indicating that SR is indeed triggered by stress, and exposure to stressful life events may increase engagement in ruminative thinking [9]. Furthermore, after accounting for the interaction between PS levels and trait anxiety, trait anxiety itself did not independently predict SR levels but rather influenced SR through its interaction with stressors. This further suggests that SR is modulated to a greater extent by how individuals perceive and react to stressors in their daily lives.

A key and illuminating finding enhances our understanding of contextual response differences: the correlations between laboratory-measured means/standard deviations of PS, SR, and NA and their corresponding EMA metrics were significant only in the LTA group and not in the HTA group. This lack of correlation in the HTA group is unlikely to be solely attributable to insufficient measurement validity, as the significant correlations in the LTA group demonstrate that the TSST laboratory paradigm can, to some extent, simulate and reflect the stress response patterns of low anxiety individuals in their daily lives. Conversely, the absence of correlation in the HTA group suggests a systematic discrepancy between this group's response to the standardized laboratory stressor and their response patterns in real-world, variable environments. This may be due to the HTA group's varying sensitivity to different types of stressors. Future research should more thoroughly explore the heterogeneity in HTA individuals' responses to stressors across dimensions such as intensity, social nature, and personal relevance.

### 4.3. Clinical Implications

The present findings offer key directions for clinical intervention in HTA populations. SR may serve as a biobehavioral marker for early identification and dynamic tracking of anxiety-susceptible individuals via wearable technologies [49]. Given the bidirectional vicious cycle between SR and NA observed here, interventions must target their reciprocal interplay. Existing research shows that cultivating mindfulness in daily life [50] and using a detached reappraisal emotion-regulation strategy [51] can effectively reduce rumination frequency and alleviate emotional distress. However, mindfulness alone cannot disrupt SR–NA coupling despite symptom alleviation, highlighting the need for supplementary attentional-control interventions. Moreover, neuroimaging methods (e.g., functional near-infrared spectroscopy) can identify abnormal activation patterns in rumination-related networks, such as in the dorsolateral prefrontal cortex and superior temporal gyrus of high trait ruminators [52], supporting individualized neuromodulation therapies. This multimodal framework integrates biomarker monitoring, cognitive–behavioral training, and neural modulation. It may transcend conventional treatment limitations to provide precision care for HTA populations.

## 5. Limitations

Our study has several limitations. One limitation is that smartphone-based EMA requires participants to be somewhat familiar with mobile devices, so the sample mainly consisted of adult university students from our institution, which may affect the generalizability of the findings. Future research should broaden the age range of the sample and develop standardized measures for assessing participants' familiarity with mobile technology [53]. In the laboratory study, the limited number of sampling time points may affect the accuracy of the results. Future research could consider increasing the sampling points in the laboratory. Another limitation is the gender imbalance in the sample, which included a relatively low proportion of male participants. Considering that gender may influence stress reactivity, subsequent studies should optimize recruitment strategies to achieve gender balance by expanding the sample size, thereby, allowing for a more in-depth exploration of the interactions among trait anxiety, SR, and gender. In terms of research dimensions, the current study focuses on the impact of SR on NA in individuals with HTA. However, other biopsychological factors—such as coping strategies and variations in cortisol responses [54], among others—may also influence the effect of SR on NA in these individuals. Future research could incorporate additional psychophysiological variables and, through multimodal data collection, construct a more comprehensive model for explaining stress responses. Since this study mainly focused on observational phenomena, further research is needed to explore intervention strategies (e.g., exposure to natural images) [55] that could reduce SR in individuals with HTA and help maintain mental health.

## 6. Conclusions

This study, by combining laboratory and daily ecological data on SR, found that SR has both state-like and trait-like characteristics, with a stronger trait-like expression seen in individuals with HTA. Further analysis revealed a vicious cycle between SR and NA in the HTA group. Breaking this cycle for individuals with HTA may be approached through interventions targeting attentional control.

## Figures and Tables

**Table 1:** Multilevel model results of lagging PS, SR (*t−n*) predicting subsequent PS, SR (*t*) (*n* = 1, 2).

	Beta coefficient	SE	*t*	*p*
Model 1: perceived stress level at time *t −* 1 (PS)				
Intercept	−1.560	18.840	−0.083	0.934
Group	−3.470	2.980	−1.166	0.244
PS level at time *t −* 1	0.480	0.058	8.355	<0.001
Gender	−2.200	3.110	−0.706	0.480
Age	1.280	0.860	1.483	0.138
Group × PS level at time *t −* 1	−0.050	0.038	−1.356	0.175
Model 2: perceived stress level at time *t −* 2 (PS)				
Intercept	−2.690	20.510	−0.131	0.896
Group	−3.060	3.330	−0.918	0.359
PS level at time *t −* 2	0.490	0.070	6.576	<0.001
Gender	−2.550	3.390	−0.753	0.451
Age	1.400	0.930	1.490	0.136
Group × PS level at time *t −* 2	−0.090	0.050	−1.697	0.090
Model 1: stress-reactive rumination at time *t −* 1 (SR)				
Intercept	286.900	179.950	1.594	0.111
Group	−46.840	29.300	−1.599	0.110
SR at time *t −* 1	0.540	0.056	9.611	<0.001
Gender	−27.190	31.440	−0.865	0.387
Age	−1.420	8.230	−0.173	0.863
Group × SR at time *t −* 1	−0.110	0.040	−2.685	0.007
Model 2: stress-reactive rumination at time *t −* 2 (SR)				
Intercept	258.490	167.640	1.542	0.123
Group	−57.810	27.540	−2.099	0.036
SR at time *t −* 2	0.530	0.070	7.658	<0.001
Gender	−21.030	28.440	−0.739	0.460
Age	−0.280	7.670	−0.036	0.971
Group × SR at time *t −* 2	−0.060	0.050	−1.142	0.254

*Note:* The reference group was the low trait anxiety group. Beta coefficients represent comparisons with this group.

**Table 2:** The autoregressive and cross-lagged parameters of SR and NA in different trait anxiety groups (EMA and Laboratory).

Group	Path	Phi coefficient	SD	*p*
EMA results				

HTA	SR_*t*−1_→SR_*t*_	0.228	0.039	<0.001
	NA_*t*−1_→NA_*t*_	0.308	0.035	<0.001
	SR_*t*−1_→NA_*t*_	0.159	0.038	<0.001
	NA_*t*−1_→SR_*t*_	0.072	0.040	0.038

LTA	SR_*t*−1_→SR_*t*_	0.118	0.035	0.001
	NA_*t*−1_→NA_*t*_	0.232	0.032	<0.001
	SR_*t*−1_→NA_*t*_	0.199	0.033	<0.001
	NA_*t*−1_→SR_*t*_	0.027	0.037	0.240

Laboratory results				

HTA	△SR_2_→△SR_3_	0.506	0.134	<0.001
	△NA_2_→△NA_3_	0.592	0.098	<0.001
	△SR_2_→△NA_3_	−0.005	0.005	0.090
	△NA_2_→△SR_3_	2.985	3.122	0.150

LTA	△SR_2_→△SR_3_	−0.345	0.253	0.050
	△NA_2_→△NA_3_	-0.624	0.193	<0.001
	△SR_2_→△NA_3_	0.003	0.006	0.350
	△NA_2_→△SR_3_	0.970	7.575	0.420

## Data Availability

All the data needed to evaluate the conclusions in the paper are present in the article and/or the Supporting Information.

## Supporting Information

Supporting Information This section comprises Tables S1–S5 and Figure S1, which are provided separately from the main manuscript. Figure S1. Correlation of the questionnaire in the laboratory and EMA. The scatterplot shows the correlations between the laboratory and EMA averages of perceived stress (a), stress-reactive rumination (b), negative affect (c), and its standard deviation (d). Red dots represent the high trait anxiety group (HTA), while blue dots represent the low trait anxiety group (LTA).

## References

[1] Baxter A. J., Vos T., Scott K. M., Ferrari A. J., Whiteford H. A. (2014). The Global Burden of Anxiety Disorders in 2010. *Psychological Medicine*.

[2] Remes O., Brayne C., van der Linde R., Lafortune L. (2016). A Systematic Review of Reviews on the Prevalence of Anxiety Disorders in Adult Populations. *Brain and Behavior*.

[3] Knowles K. A., Olatunji B. O. (2020). Specificity of Trait Anxiety in Anxiety and Depression: Meta-Analysis of the State-Trait Anxiety Inventory. *Clinical Psychology Review*.

[4] Weger M., Sandi C. (2018). High Anxiety Trait: A Vulnerable Phenotype for Stress-Induced Depression. *Neuroscience & Biobehavioral Reviews*.

[5] Watkins E. R. (2008). Constructive and Unconstructive Repetitive Thought. *Psychological Bulletin*.

[6] Wu L., Lin B. X., Liu L. (2017). Difficulties of Highly Anxious Individuals in Removing Attention From Emotionally Negative Information. *Journal of Psychological Science*.

[7] Rood L., Roelofs J., Bögels S. M., Alloy L. B. (2010). Dimensions of Negative Thinking and the Relations With Symptoms of Depression and Anxiety in Children and Adolescents. *Cognitive Therapy and Research*.

[8] Nolen-Hoeksema S. (2000). The Role of Rumination in Depressive Disorders and Mixed Anxiety/Depressive Symptoms. *Journal of Abnormal Psychology*.

[9] Michl L. C., McLaughlin K. A., Shepherd K., Nolen-Hoeksema S. (2013). Rumination as a Mechanism Linking Stressful Life Events to Symptoms of Depression and Anxiety: Longitudinal Evidence in Early Adolescents and Adults. *Journal of Abnormal Psychology*.

[10] Mogg K., Bradley B. P. (2018). Anxiety and Threat-Related Attention: Cognitive-Motivational Framework and Treatment. *Trends in Cognitive Sciences*.

[11] Cho S., White K. H., Yang Y., Soto J. A. (2019). The Role of Trait Anxiety in the Selection of Emotion Regulation Strategies and Subsequent Effectiveness. *Personality and Individual Differences*.

[12] Elwood L. S., Wolitzky-Taylor K., Olatunji B. O. (2012). Measurement of Anxious Traits: A Contemporary Review and Synthesis. *Anxiety, Stress & Coping*.

[13] Stone A. A., Shiffman S. (1994). Ecological Momentary Assessment (EMA) in Behavorial Medicine. *Annals of Behavioral Medicine*.

[14] Funk J., Müller C., Rosenkranz T. (2025). An Ecological Momentary Assessment Study Assessing Repetitive Negative Thinking as a Predictor for Psychopathology. *PLoS One*.

[15] Zheng X. Z., Wang W. L., Zhou Y. Q. (2021). Application Progress of Ecological Momentary Assessment in Field of Mental Disorders. *Chinese Nursing Research*.

[16] Zhu X., Yang Y., Xiao Z. (2024). Daily Life Affective Dynamics as Transdiagnostic Predictors of Mental Health Symptoms: an Ecological Momentary Assessment Study. *Journal of Affective Disorders*.

[17] Clark V., Kim S. J. (2021). Ecological Momentary Assessment and mHealth Interventions Among Men Who Have Sex With Men: Scoping Review. *Journal of Medical Internet Research*.

[18] Laicher H., Int-Veen I., Torka F. (2022). Trait Rumination and Social Anxiety Separately Influence Stress-Induced Rumination and Hemodynamic Responses. *Scientific Reports*.

[19] Rosenbaum D., Thomas M., Hilsendegen P. (2018). Stress-Related Dysfunction of the Right Inferior Frontal Cortex in High Ruminators: An fNIRS Study. *NeuroImage: Clinical*.

[20] Kirschbaum C., Pirke K.-M., Hellhammer D. H. (2004). The Trier Social Stress Test–a Tool for Investigating Psychobiological Stress Responses in a Laboratory Setting. *Neuropsychobiology*.

[21] Rosenbaum D., Hilsendegen P., Thomas M. (2018). Disrupted Prefrontal Functional Connectivity During Post-Stress Adaption in High Ruminators. *Scientific Reports*.

[22] Gianferante D., Thoma M. V., Hanlin L. (2014). Post-Stress Rumination Predicts HPA Axis Responses to Repeated Acute Stress. *Psychoneuroendocrinology*.

[23] Zoccola P. M., Dickerson S. S. (2012). Assessing the Relationship Between Rumination and Cortisol: A Review. *Journal of Psychosomatic Research*.

[24] Gotlib I. H., McLachlan A. L., Katz A. N. (1988). Biases in Visual Attention in Depressed and Nondepressed Individuals. *Cognition & Emotion*.

[25] Wang Z., Yuan C.-M., Huang J. (2011). Reliability and Validity of the Chinese Version of Beck Depression Inventory-II Among Depression Patients. *Chinese Mental Health Journal*.

[26] Zheng X. H., Xu D., Shu L. (1993). A Test Report on State–trait Anxiety in Changchun. *Chinese Mental Health Journal*.

[27] Pan D.-N., Hoid D., Wang X.-B., Jia Z., Li X. (2022). When Expanding Training From Working Memory to Emotional Working Memory: Not Only Improving Explicit Emotion Regulation but Also Implicit Negative Control for Anxious Individuals. *Psychological Medicine*.

[28] Li W., Liu S., Han S., Zhang L., Xu Q. (2022). Emotional Bias of Trait Anxiety on Pre-Attentive Processing of Facial Expressions: ERP Investigation. *Acta Psychologica Sinica*.

[29] Zeng J. J., Jiang T., Chen Q. L., Hu S. Y., Chen Y. (2021). An Event- Related Potentials Study of Visual and Auditory Information Processing in Young People With Different Trait Anxiety. *Chinese Mental Health Journal*.

[30] Hoebeke Y., Blanchard M. A., Contreras A., Heeren A. (2022). An Experience Sampling Measure of the Key Features of Rumination. *Clinical Neuropsychiatry*.

[31] Xiao J., Zhu X. Z., Yao S. Q., Ling Y., JRZ A., ARP A. (2008). The Reliability and Validity of Chinese Translation of RSQ-SSV in Undergraduate Student. *Chinese Mental Health Journal*.

[32] Watson D., Clark L. A., Tellegen A. (1988). Development and Validation of Brief Measures of Positive and Negative Affect: The PANAS Scales. *Journal of Personality and Social Psychology*.

[33] Strahler J., Skoluda N., Kappert M. B., Nater U. M. (2017). Simultaneous Measurement of Salivary Cortisol and Alpha-Amylase: Application and Recommendations. *Neuroscience & Biobehavioral Reviews*.

[34] Asparouhov T., Hamaker E. L., Muthén B. (2018). Dynamic Structural Equation Models. *Structural Equation Modeling: A Multidisciplinary Journal*.

[35] Hamaker E. L., Asparouhov T., Brose A., Schmiedek F., Muthén B. (2018). At the Frontiers of Modeling Intensive Longitudinal Data: Dynamic Structural Equation Models for the Affective Measurements From the COGITO Study. *Multivariate Behavioral Research*.

[36] Hjartarson K. H., Snorrason I., Bringmann L. F., Ólafsson R. P. (2022). Automaticity and Depression: Daily Mood-Reactive Rumination in People With and Without Depression History. *Journal of Psychopathology and Clinical Science*.

[37] Schuurman N. K., Ferrer E., de Boer-Sonnenschein M., Hamaker E. L. (2016). How to Compare Cross-Lagged Associations in a Multilevel Autoregressive Model. *Psychological Methods*.

[38] Rosenbaum D., Int-Veen I., Laicher H. (2021). Insights From a Laboratory and Naturalistic Investigation on Stress, Rumination and Frontal Brain Functioning in MDD: An fNIRS Study. *Neurobiology of Stress*.

[39] Rosenbaum D., Int-Veen I., Kroczek A. (2020). Amplitude of Low Frequency Fluctuations (ALFF) of Spontaneous and Induced Rumination in Major Depression: An fNIRS Study. *Scientific Reports*.

[40] Eysenck M. W., Derakshan N., Santos R., Calvo M. G. (2007). Anxiety and Cognitive Performance: Attentional Control Theory. *Emotion*.

[41] Su-Hao P., Qian T., Bin X. (2014). Inhibitory Control on Induced Negative Emotion in Individuals With Trait Anxiety. *Chinese Mental Health J*.

[42] Zhang L.-W. (2015). Competitive Trait Anxiety Interferes With Inhibition Function: Examination of Attentional Control Theory. *Journal of Psychological Science*.

[43] Whitmer A. J., Gotlib I. H. (2013). An Attentional Scope Model of Rumination. *Psychological Bulletin*.

[44] Corr R., Pelletier-Baldelli A., Glier S., Bizzell J., Campbell A., Belger A. (2021). Neural Mechanisms of Acute Stress and Trait Anxiety in Adolescents. *NeuroImage: Clinical*.

[45] Glier S., Campbell A., Corr R., Pelletier-Baldelli A., Belger A. (2022). Individual Differences in Frontal Alpha Asymmetry Moderate the Relationship Between Acute Stress Responsivity and State and Trait Anxiety in Adolescents. *Biological Psychology*.

[46] Souza G. G. L., Mendonça-de-Souza A. C. F., Duarte A. F. A. (2015). Blunted Cardiac Reactivity to Psychological Stress Associated With Higher Trait Anxiety: A Study in Peacekeepers. *BMC Neuroscience*.

[47] Robinson M. S., Alloy L. B. (2003). Negative Cognitive Styles and Stress-Reactive Rumination Interact to Predict Depression: A Prospective Study. *Cognitive Therapy and Research*.

[48] Rood L., Roelofs J., Bögels S. M., Meesters C. (2012). Stress-Reactive Rumination, Negative Cognitive Style, and Stressors in Relationship to Depressive Symptoms in Non-Clinical Youth. *Journal of Youth and Adolescence*.

[49] Arnold V. X., Young S. D. (2025). The Potential of Wearable Sensors for Detecting Cognitive Rumination: A Scoping Review. *Sensors*.

[50] Bolzenkötter T., Neubauer A. B., Koval P. (2025). Impact of a Momentary Mindfulness Intervention on Rumination, Negative Affect, and Their Dynamics in Daily Life. *Affective Science*.

[51] Zhu L., Fu T., Yan X., Yuan J., Yang J. (2024). The Neurophysiological Effects of Detached and Positive Reappraisal During the Regulation of Self-Conscious Emotions. *Psychoradiology*.

[52] Long J., Peng L., Li Q. (2024). Acute Stress Impairs Intentional Memory Suppression Through Aberrant Prefrontal Cortex Activation in High Trait Ruminators. *International Journal of Clinical and Health Psychology*.

[53] Liu H., Lou V. W. (2019). Developing a Smartphone-Based Ecological Momentary Assessment Protocol to Collect Biopsychosocial Data With Community-Dwelling Late-Middle-Aged and Older Adults. *Translational Behavioral Medicine*.

[54] Villada C., Hidalgo V., Almela M., Salvador A. (2016). Individual Differences in the Psychobiological Response to Psychosocial Stress (Trier Social Stress Test): The Relevance of Trait Anxiety and Coping Styles. *Stress and Health*.

[55] Michels N., Debra G., Mattheeuws L., Hooyberg A. (2022). Indoor Nature Integration for Stress Recovery and Healthy Eating: A Picture Experiment With Plants Versus Green Color. *Environmental Research*.

