# Supplementary material for: Bidirectional Dynamics Between Stress-Reactive Rumination and Negative Affect: Evidence From a Multimethods Study in Individuals With High Trait Anxiety
Source: Depress Anxiety. 2025 Sep 2;2025:2503361. doi: 10.1155/da/2503361 (PMC12419933; doi:10.1155/da/2503361)
Supplement: Supporting Information — This section comprises Tables S1–S5 and Figure S1, which are provided separately from the main manuscript. Figure S1. Correlation of the questionnaire in the laboratory and EMA. The scatterplot shows the correlations between the laboratory and EMA averages of perceived stress (a), stress-reactive rumination (b), negative affect (c), and its standard deviation (d). Red dots represent the high trait anxiety group (HTA), while blue dots represent the low trait anxiety group (LTA). [file 2503361.f1.docx]

***Supplementary***

**Bidirectional Dynamics Between Stress-reactive Rumination and Negative Affect: Evidence from a Multi-methods Study in Individuals with High Trait Anxiety**

Zhu Qingzi^1+^, Peng Lanxin^1+^, Niu Lijing^1^, Zeng Yuanyuan^1^, Chen Xiayan^1^, Chen Zini^1^, Dai Haowei^1^, Zhang Ruibin^1,2^

^1^Laboratory of Cognitive Control and Brain Healthy, Department of Psychology, School of Public Health, Southern Medical University, Guangzhou, PRC China

^2^Center for Brain Science and Brain-Inspired Intelligence, Guangdong-Hong Kong-Macao Greater Bay Area

^3^Department of Psychiatry, Zhujiang Hospital, Philosophy Doctor, Southern Medical University, Guangzhou, PRC China

+These authors contributed equally to this work.

Correspondence and request for materials should be addressed to:

Ruibin Zhang, Laboratory of Cognitive Control and Brain Healthy, Department of Psychology, School of Public Health, Southern Medical University, E-mail: [ruibinzhang@foxmail.com](mailto:ruibinzhang@foxmail.com)

**Table S1 Stress-reactive rumination items and laboratory-based multidimensional stress items.**

| **Dimension** | **Items for the assessment of stress-reactive rumination** |
| --- | --- |
| Rehearsal | 1. I keep thinking about what I said or did when the event happened. |
|  | 2. I keep thinking about how I performed in similar past situations. |
| Intrusion | 3. I cannot stop thinking about the causes and consequences of the event. |
|  | 4. I keep wondering why similar events always happen to me. |
| Reflection | 5. I think about why I reacted the way I did in this event. |
|  | 6. I think about what this event means to me. |
| Self-Criticism | 7. I think about why I couldn’t handle the event better. |
|  | 8. I think about why I couldn’t respond in a better way to this event. |
| Negativity | 9. This event makes it hard for me to stop thinking negatively about myself. |
|  | 10. I think about the negative impact this event will have on me. |
|  | **Items for laboratory-based multidimensional stress** |
| Perceived Stress | Please assess the level of stress you are currently perceiving. |
| Unpleasantness | Please assess the level of unpleasantness you are currently perceiving. |
| Difficulty | Please assess the level of difficulty you are currently perceiving. |
| Irritation | Please assess the level of irritation you are currently perceiving. |
| Fear | Please assess the level of fear you are currently perceiving. |

**Table S2 Demographic information and scale scores.**

|  | **HTA (*n*=31)** | | | **LTA(*n*=31)** | | | ***t/χ^2^*** | ***p*** |
| --- | --- | --- | --- | --- | --- | --- | --- | --- |
|  | | **Mean** | **SD** | | **Mean** | **SD** |  |  |
| Age | | 21.900 | 1.535 | | 22.060 | 1.504 | -0.418 | 0.678 |
| Gender(M/F) | | 7/24 |  | | 11/20 |  | 1.253 | 0.263 |
| STAI-T | | 52.480 | 2.393 | | 28.550 | 3.394 | 32.089 | <0.001 |
| PHQ-9 | | 7.230 | 2.963 | | 2.770 | 2.217 | 6.698 | <0.001 |
| BDI-II | | 9.550 | 3.948 | | 2.740 | 2.804 | 7.825 | <0.001 |

Note: M = Male, F = Female, STAI-T = State-Trait Anxiety Inventory–Trait subscale, PHQ–9 = Patient Health Questionnaire-9, BDI-II = Beck Depression Inventory II, HTA = High Trait Anxiety, LTA = Low Trait Anxiety.

**Table S3 Differences in EMA scores between high and low trait anxiety groups.**

| **Group** | **HTA (*n*=31)** | | **LTA(*n*=31)** | | ***t*** | ***p*** |
| --- | --- | --- | --- | --- | --- | --- |
|  | **Mean** | **SD** | **Mean** | **SD** |  |  |
| PS | 35.071 | 25.666 | 26.408 | 23.639 | 10.105 | <0.001 |
| PSI | 38.562 | 27.676 | 30.807 | 25.184 | 8.435 | <0.001 |
| SR | 308.444 | 245.767 | 183.280 | 188.223 | 16.458 | <0.001 |
| NA | 16.842 | 6.004 | 14.216 | 4.831 | 13.868 | <0.001 |

Note: PS = Perceived Stress, PSI = Perceived Stress Impact, SR = Stress-reactive Rumination, NA = Negative Affect

**Table S4 Multilevel model results of group predicting PS and SR, group and PS predicting SR.**

| **Multilevel model results of group predicting PS and SR** | | | | | | |
| --- | --- | --- | --- | --- | --- | --- |
| **Dependent Variable** | |  | **Beta Coefficient** | **SE** | ***t*** | ***p*** |
| **SR** | **Fixed Effects** | |  |  |  |  |
|  | Intercept | | 496.050 | 248.830 | 1.994 | 0.046 |
|  | Group | | -117.870 | 43.980 | -2.680 | 0.007 |
|  | Gender | | -47.180 | 49.440 | -0.954 | 0.340 |
|  | Time | | 2.410 | 2.230 | 1.081 | 0.280 |
|  | Age | | -0.840 | 11.220 | -0.075 | 0.940 |
|  | **Random Effects** | |  |  |  |  |
|  | Intercept | | 28991.680 | 17.030 |  |  |
|  | Residual | | 20161.990 | 18.160 |  |  |
| **PS** | **Fixed Effects** | |  |  |  |  |
|  | Intercept | | 17.920 | 27.200 | 0.659 | 0.510 |
|  | Group | | -8.390 | 4.420 | -1.899 | 0.058 |
|  | Gender | | -3.350 | 4.990 | -0.673 | 0.501 |
|  | Time | | 0.500 | 0.280 | 1.751 | 0.080 |
|  | Age | | 1.290 | 1.290 | 1.044 | 0.297 |
|  | **Random Effects** | |  |  |  |  |
|  | Intercept | | 290.160 | 17.030 |  |  |
|  | Residual | | 329.630 | 18.160 |  |  |
| **Multilevel model results of group and PS predicting SR** | | | | | | |
| **SR** | **Fixed Effects** | |  |  |  |  |
|  | Intercept | | 339.820 | 159.230 | 2.134 | 0.033 |
|  | Group | | -12.210 | 26.620 | -0.459 | 0.646 |
|  | Pressure | | 8.110 | 0.300 | 26.681 | <0.001 |
|  | Gender | | -29.400 | 29.220 | -1.006 | 0.314 |
|  | Time | | -0.060 | 1.650 | -0.034 | 0.973 |
|  | Age | | -8.920 | 7.240 | -1.232 | 0.218 |
|  | Group*Pressure | | -2.060 | 0.210 | -10.049 | <0.001 |
|  | **Random Effects** | |  |  |  |  |
|  | Intercept | | 9973.390 | 99.870 |  |  |
|  | Residual | | 11026.190 | 105.010 |  |  |

Note: The reference group was the low trait anxiety group. Beta coefficients represent comparisons with this group.

**Table S5 ANOVA results of the differences in subjective questionnaire scores between high and low trait anxiety groups under different time points.**

| **Questionnaire Measures** | ***F*_（1,55）_** | ***p*** |
| --- | --- | --- |
| ***LMS Total Score***  Time Point  Group  Time Point × Group  ***Laboratory perceived stress***  Time Point  Group  Time Point × Group  ***SR***  Time Point  Group  Time Point × Group  ***NA***  Time Point  Group  Time Point × Group  ***Cortisol Concentration***  Time Point  Group  Time Point × Group | 28.763  0.380  1.408  18.212  0.647  3.348  40.189  3.625  0.137  30.513  6.604  0.705  66.026  57.984  5.091 | <0.001  0.540  0.249  <0.001  0.425  0.043*****  <0.001  0.062  0.872  <0.001  0.013  0.499  <0.001  <0.001  0.001 |

Note: LMS = Laboratory-based Multidimensional Stress

**
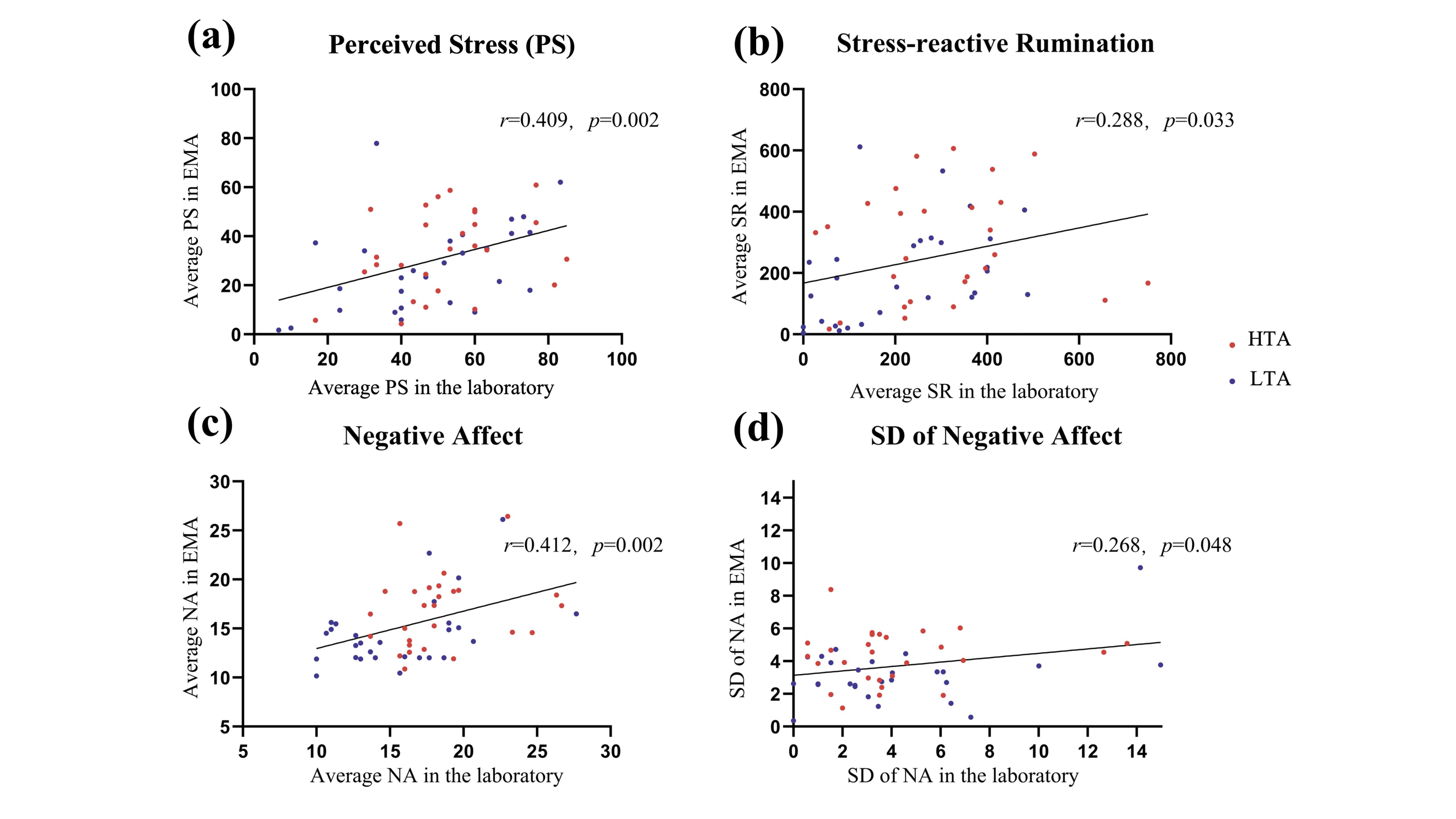
**

**Fig. S1 Correlation of the questionnaire in laboratory and EMA.** The scatterplot shows the correlations between the laboratory and EMA averages of perceived stress (a), stress-reactive rumination (b), negative affect (c), and its standard deviation (d). Red dots represent the high-trait anxiety group (HTA), while blue dots represent the low trait anxiety group (LTA).
